# Traditional bone setting and trauma care in South-Eastern Chad

**DOI:** 10.4314/ahs.v22i2.70

**Published:** 2022-06

**Authors:** Canaan J Hancock, Hissein Breme, Jan P Mazur, Keerthana Chintalapati, Andrew Hashikawa, Eric Kroner

**Affiliations:** 1 Washington University in St. Louis, Department of Anthropology; 2 EcoleNormaleSuperieure N'Djamena; 3 University of Michigan Medicine, Department of Emergency Medicine; 4 TEAM Chad

**Keywords:** Wounds And Injuries, African Traditional, Orthopedics

## Abstract

**Background:**

Traditional bonesetters are the main providers of fracture treatment and trauma care in much of Africa. However, there is a paucity of literature on bonesetters in Chad.

**Objectives:**

Our study sought to investigate Chadian bonesetter practices, their relationship to the community, and the complex local perspectives on trauma care in Am Timan, Chad.

**Methods:**

Thirty-three semi-structured interviews were conducted with community members, traditional bonesetters, and physicians in Am Timan using a constructivist grounded theory approach. Responses were coded, categorized, and compared within and across study populations to identify themes.

**Results:**

Most community members (n=25) interviewed preferred bonesetters for trauma care due to their affordability, continuity and convenience of care, and the community's fear of Western medical practices. Although the Chadian bonesetters' fracture management mirrored bonesetters in neighboring African countries, the Chadian bonesetters have a much wider scope of practice, including treatment for both medical and spiritual ailments. Both Jabari (n=6) and physicians (n=2) emphasized the need for more training and collaboration.

**Conclusion:**

As in much of Africa, bonesetters perform a major role in providing trauma care in Chad. Our research identifies an opportunity to maximize trauma care in Chad through dialogue, training, and collaboration between bonesetters and physicians.

## Introduction

Traditional bonesetters (TBS) are a form of traditional healers specializing in musculoskeletal (MSK) injuries, and they have long been trusted providers in sub-Saharan communities^1^–^4^. Trauma care in many parts of Africa is carried out by traditional bonesetters, even in urban areas with available government hospitals^5^–^11^. This may be due to issues plaguing healthcare systems such as lack of medical staff, lack of transportation to healthcare facilities, and inequitable allocation of government funding^6^,^9^,^12^–^14^. Many African communities may also prefer TBS over Western medicine due to their convenience of care, affordability, fear of Western medical practices, and preference for traditional remedies^1^,^4^,^13^,^15^. Previous studies have found that local physicians understand the importance of TBS to the healthcare system^3^.

However, numerous studies in sub-Saharan Africa have documented complications arising from treatment by TBS^6^,^10^,^16^–^18^. In some African countries, lack of formal training and regulation leads to inexperienced and ill-equipped TBS being able to practice without safety or outcome surveillance. In response, there have been growing calls from the medical community for TBS to be trained, regulated, and integrated into existing healthcare systems, supported by successful short training programs seen in Nigeria and Ethiopia^4^,^8^,^9^,^15^–^17^,^19^,^20^. However, no substantial implementation of these ideas have been attempted yet in sub-Saharan Africa.

While the medical community and governments in other African countries have been working to study and integrate TBS into their healthcare systems, there is currently a paucity of data available on TBS in the country of Chad. Chadian bonesetters, locally known as Jabari, typically do not have formalized training, regulation, or referral systems, and little is known about their scope of practice, or their relationships with each other, the community, and the government healthcare system in Chad. Our study aimed to explore Jabari practices, their relationship to the community, and the complex local perspectives on trauma care in the Salamat Region of southeastern Chad by interviewing three different stakeholder groups from five neighborhoods within Am Timan. Obtaining local Chadian perspectives directly from community citizens, Jabari, and government healthcare providers are needed to better inform potential barriers to developing interventions that can leverage the largely underutilized trauma care Jabari work force.

## Methods

### Study Setting

The Salamat Region of Chad was designated the “poorest region in the world” in 2015 by the Oxford Poverty and Human Development Initiative, with 98% of the population living in poverty^21^. Trauma care is both expensive and limited in Chad, with very few specialists for orthopedic trauma^22^,^23^. The study took place in Am Timan, the capital of the Salamat Region and the sixth largest city in Chad, with approximately 50,000 people. A small regional hospital that provides limited trauma care operates within the city.

### Study Participants

In July 2018, community members were invited to participate using cluster and opportunity sampling. At least four randomly selected people in each neighborhood were interviewed in each of the five major neighborhoods, representing five clusters, within Am Timan. Within neighborhoods, we used opportunity sampling to enroll participants, given the lack of recent census or community registration. To provide sufficient data for thematic analysis, we wanted at least 20 community member participants. Traditional bonesetters and physicians were identified using purposive sampling, with no minimum number due to the limited number of both groups in the region. Age and years of experience were not considered when sampling traditional bonesetters or physicians. All interviews wre conducted in-person. Neither investigator that participated in interviews had prior relationships with any participants.

### Study Design

Our study used a semi-structured interview guide while allowing for tangential responses. The guide was written in English, then translated into Chadian Arabic and French by one of the investigators, a native speaker proficient in English, French, and Chadian Arabic (HB). Several additional native speakers were also consulted during translation to pretest our questions for reliability and validity. The interview guide was not back translated into English due to a lack of other available English-speaking translators. We used a constructivist grounded theory approach to examine different perspectives amongst Jabari, government healthcare providers, and community citizens on trauma care in Am Timan. Each subsequent interview was iteratively informed by previous interviews to elucidate themes that developed. Additional questions were added to evaluate and expand on these emerging themes.

### Data Collection

Interviews were conducted by a native speaker and an English speaker proficient in Chadian Arabic. Community participants were approached in person, while Jabari and physicians were contacted by telephone prior to meeting in-person for the interview. Each interview began with investigators reading a research information sheet to gain verbal consent and ensure participants were interviewed in their preferred language. Investigators then interviewed participants with the guide, while also allowing the participant to direct the conversation to other related topics. The interview guide initially consisted of six questions:

1. What do you consider to be trauma?
2. What kinds of trauma do you see most often?
3. Where do you see the most trauma in your community?
4. What do people in Am Timan think are the best options for trauma care? How do they decide which healthcare providers to see?
5. How do the different healthcare providers in Am Timan treat traumatic injuries?
6. Who are the key community stakeholders in healthcare in AmTiman? What are their roles?

Additional follow-up questions were used so participants could elaborate on their responses. Interviews were recorded on a digital voice recorder. Responses were erased after being transcribed. Identifying information (e.g. names and addresses) were expunged.

### Data Analysis

**Figure 1:**
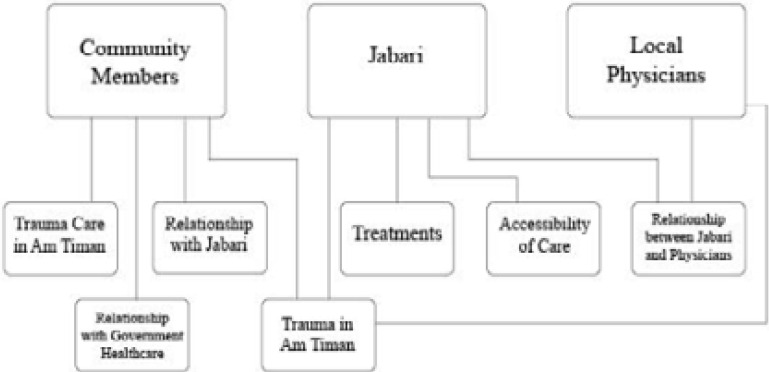
Interview Response Categories

We iteratively applied thematic content analysis to the transcribed interviews using three investigators following translation of the interviews into English from Chadian Arabic and French. Responses were reviewed through open coding to elucidate all potential themes, then codes were compared to identify common categories. Authors used axial coding to place responses into categories (Figure 1), which were compared within and across study populations to find emerging themes. The consolidated criteria for reporting qualitative research (COREQ) checklist^24^, was used to inform the study design, analysis, and reporting process.

## Results

### Trauma in Am Timan

Twenty-seven community members were invited to be interviewed, and twenty-five of them agreed to participate. The community members had an average age of 36, with a range from 24 to 55 years of age. Due to cultural considerations that limits male-female interactions outside of marriage, only four females participated in interviews. Six Jabari and three physicians were invited to participate, and all six Jabari and two of the physicians agreed to participate. To our knowledge, all Jabari and physicians in this region were males, so no female Jabari or physicians were interviewed.

Two options for trauma care emerged: care by Jabari or the local hospital. Both options seemed to contain numerous challenges for patients and providers. As reported by six community members, both physicians, and four Jabari, the hospital and Jabari frequently struggled with medical supply shortages. Rural areas surrounding Am Timan suffered from a near-complete lack of medical supplies, and during the rainy season, travel to any city healthcare provider from these rural areas was “very difficult” according to community members and Jabari. The physicians, Jabari, and six community members also mentioned that the hospital lacked orthopedic specialists and the technology required to adequately evaluate trauma.

### Jabari Perspectives

See Table 1 for reported ages, education, and years of practice by the six Jabari interviewed.

**Table 1:** Jabari Demographics

	*Age*	*Education*	*Training*	*Years of Experience*
Jabari #1	48	NFE	Family	25
Jabari #2	49	NFE	Spiritual Guidance	30
Jabari #3	58	NFE	Family	36
Jabari #4	33	NFE	Family	15
Jabari #5	45	Primary School	Family	21
Jabari #6	43	Primary School	Family	-

*NFE = No Formal Education

#### Treatments

**Image 1:**
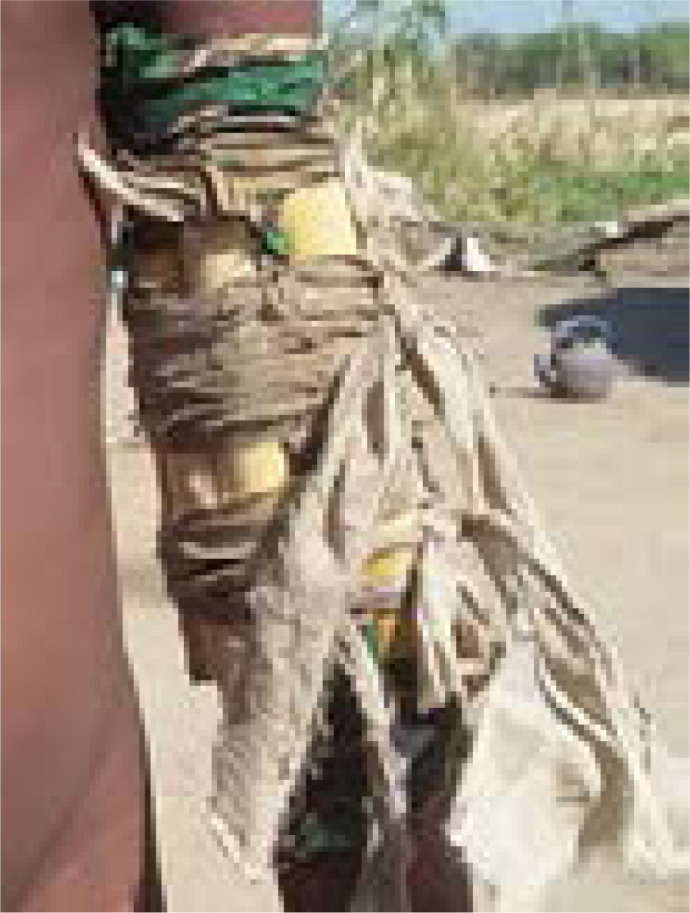
A Jabari splint made from cloth and small sticks bound together. Bonesetters in many African countries use a similar technique

Each Jabari stated they treated all types of fractures, including spinal, pelvic, and open fractures, and all had similar fracture treatment methods. Manual manipulation techniques were used to reduce the fracture to anatomical position, then they applied an herbal cream locally known as “Promate”. The fracture site was then wrapped in cloth and splinted with bound small sticks (Image 1). If the injury site did not heal properly, the bonesetters broke the bone again and repeated the previous process. For open fractures, the Jabari used the same process while adding antibiotic ointment and bleeding control treatments.

All six Jabari stated they also cared for other forms of trauma. “Fahiiq”, mentioned by each Jabari, was said to be a form of trauma resulting from head or neck injuries that cause blood to build up in the trachea and nasal passages. They all reportedly treated “fahiiq” by puncturing the palate to drain the blood out of the nasal cavities. Blood-letting is considered the preferred treatment method for muscular injuries^25^, while black stones were used to draw out snake venom after a tourniquet has been applied above snakebites.

The Jabari also stated they performed medical procedures. For example, five of the Jabari said they regularly removed the entire uvula from pediatric patients to treat vomiting (Jabari #2-6). Additionally, they performed circumcisions, teeth removal, wart removal, and other procedures that the Jabari did not elaborate on further. Bloodletting for illnesses such as hepatitis was common, although the procedure could differ from the form used on muscular injuries. For muscular injuries, five of the Jabari stated that the procedure consisted of a series of small cuts over the injury site, then a suction cup or cotton was used to draw out the blood (Jabari #1, #3-6). For illnesses, a needle was used to draw out the blood in the radial artery of the wrist, according to one of the Jabari (Jabari #2).

### Accessibility of Care

The Jabari took pride in caring for their community and making their care accessible to anyone. Five of the bonesetters stated that they traveled to patients' homes for treatment and visited the patients throughout the recovery process if the patient could not move easily. Additionally, all but one Jabari reported that they reduced prices for low-income patients. Two of these five stated they accepted “whatever patients can pay regardless of the treatment”, while the other three said they charged set rates for fractures, but lowered prices for other treatments.

### Relationship Between Jabari and Government Healthcare

Our interviews also explored overlap of care and the relationships between Jabari and local government healthcare workers. First, all six Jabari stated that they had worked with local physicians. However, four of the six Jabari stated that they were not currently allowed to work with the hospital, while the other two stated that they rarely get to assist with fractures at the hospital. One Jabari said that currently, “*some of the doctors in the hospital are new and don't trust Jabari, so [Jabari] don't get the opportunity to help in the hospital as often as they used to*” (Jabari #1).

Jabari perspectives varied with regard to training and supply-sharing with the hospital. Four out of the six Jabari interviewed stated that they had received medical supplies from government healthcare services in the past, but that they no longer received these supplies (Jabari #2-5). One Jabari emphasized the need for more trauma care supplies, especially in rural areas, but he explained the hospital itself faces a shortage of supplies (Jabari #2). Two of the interviewed Jabari stated that they had previously trained doctors and Red Cross personnel in bonesetting. However, in recent years, the training had stopped (Jabari #1, #4). When asked if they would be willing to participate in a training program by orthopedists, all of the Jabari stated that they would be willing to participate, but five of the six Jabari maintained that training should consist of equal exchange of information between Jabari and physicians.

Two physicians interviewed in this study also stated that collaboration was needed in this region. One physician stated that “*to improve the relationship between doctors and Jabari, the doctors should have training with Jabari to show them what is healthy and what's not. I want us to work together with the victims*.” This physician said that Jabari should only care for fractures, but that physicians “*can't allow other treatments like [treatment for] fahiiq and blood-letting because [the physicians] see it as harmful and can cause tetanus or a host of other infections*.” Additionally, the physician made it clear he believed that Jabari needed further training in treating fractures based on the complications from Jabari treatment that he had seen in the hospital. The physicians reported seeing poorly reduced fractures, gangrenous limbs, and serious infections from Jabari medical procedures.

### Community Members' Perspectives

Several themes emerged from the 25 community members regarding the community's relationships with the Jabari (Table 2) and government healthcare (Table 3), and their ideas on how to improve trauma care in Am Timan (Table 4). However, a few interviewees acknowledged that in the rural areas, any medical services are much more difficult to access, so people living outside of Am Timan, who we were unable to interview, may have different views and experiences with Jabari and government healthcare services.

**Table 2:** Community Member's Perspectives on Jabari

*Themes*	*Quotes*
Lower costs, n=7	“People would prefer the hospital if they had money, but Jabari are much cheaper”
Conscientiousness, n=11	“The hospital oftentimes will just give you medicine that makes you feel good for a little bit but it doesn't get rid of the problem. Jabari treat you for days and weeks on end, and they make an active effort to come check on you frequently.”
Superior treatment of MSK injuries, n=19	“Jabari actually work on bones, moving them around, pushing and pulling until the bone is set straight again”
	“The doctors don't have an orthopedist, so they just pour the plaster on the leg. Many people don't want the plaster because they put it on too tight, and they later have to amputate the leg. The Jabari know better than the doctors as to how to fix the bone.”

**Table 3:** Community Members' Perspectives on Government Healthcare

*Themes*	*Quotes*
Lack of concern for patients, n=13	“Doctors just look at the fracture, then cover it in plaster and never come check on you”
	“In the hospital, the doctors do not treat the patients well. They yell at the patients and do not take enough time with them to give proper care.”
	“In the hospital, it is not like western hospitals. The nurses do not bathe or feed the patients. It is up to the person that brings the patient to the hospital to wash them, give them clean clothes, feed them, and purchase the medications for the patient. This places a lot of burden on people and makes them not want to take people to the hospital.”
Non-MSK injuries and illnesses, n=12	“If people can afford it, they go to the hospital for everything other than fractures.”
	“If someone has a fracture, they go to Jabari. For pretty much every other issue they go to the hospital.”

**Table 4:** Trauma Care in Am Timan

*Themes*	*Quotes*
More Jabari & Physicians, n=12	“Am Timan simply needs more ambulances, doctors, and Jabari. They each have their place in helping the people.”
Collaboration between Jabari and Physicians, n=4	“The community needs both Jabari and doctors; they each are good at their specialty. To improve healthcare in Am Timan, they need more specialists like Jabari and doctors to train other people.”
	“To improve healthcare, there needs to be more specialists, and the Jabari should get trained by the specialists to make them more effective.”

## Discussion

Many key lessons can be drawn from this exploratory study regarding the Jabari in southeastern Chad. Jabari are similar to other sub-Saharan TBS with regards to many of the reasons community members patronize Jabari, the community's perception they are less expensive and more effective with fracture care, and their actual methods of fracture care^1^,^2^,^12^,^13^,^15^–^17^. This would make the Jabari seem to be ideal candidates for similar traditional bonesetter training and incorporation into government healthcare systems as called for in Nigeria, Ethiopia, and Ghana^4^,^8^,^15^,^17^.

However, one can clearly distinguish the Jabari from traditional bonesetters in nearby countries based on their scope of practice. In all the previously mentioned studies on traditional bonesetters in sub-Saharan Africa, none of the bonesetters were found to treat anything other than MSK injuries. Other forms of traditional healers treat medical illnesses in Nigeria and Ethiopia, but the bonesetters in these nations only practiced within their specialty of traumatic injury^8^,^9^,^26^. Conversely, Jabari provided medical treatments and procedures for vomiting, fever, skin lesions, circumcisions, and other medical issues, in addition to bonesetting. Many of these procedures were done without antiseptics or pain medications and reportedly led to numerous complications and infections.

In addition to Jabari being distinct from other traditional bonesetters in sub-Saharan Africa, the Chadian healthcare system is not currently equipped to handle a comparable structure of training, regulation, and referral as is being attempted in neighboring Nigeria^3^,^8^,^9^. Nigeria has approximately 400 orthopedic surgeons^3^, numerous tertiary trauma centers, and over 4,000 X-ray machines^27^, meaning the government healthcare services can feasibly train bonesetters in the use of X-ray machines and more readily regulate their practices. Meanwhile, Chad has very few orthopedic surgeons^22^, few X-ray machines outside of Chad's capital, and less resources to regulate the Jabari.

Despite these challenges, the Chadian government cannot and should not ignore or ban the Jabari. Both community members and local physicians in Am Timan understood the importance of the Jabari to trauma care in the region. In addition to the aforementioned reasons that communities in other African countries prefer TBS, community members in Am Timan appreciated the continuity of care and consistent efforts of Jabari to heal them compared to the local physicians. It is unlikely that community members would stop utilizing the Jabari for fracture care even if they were prohibited. The Jabari are a vital resource that, if effectively integrated into the Chadian healthcare system, can diminish the burden on government healthcare services, especially given their lack of orthopedists and technology.

However, more research should be done to explore how to integrate Jabari into the government healthcare system to reduce complications from Jabari treatments and encourage collaboration. We propose a two-pronged strategy to initiate integration. First, since the Jabari interviewed in this study were all willing to undergo training, orthopedic surgeons could be brought in to conduct one-day training programs as seen in Ethiopia and Nigeria^17^,^20^. These training programs found significant reductions in amputations and other complications from the trained bonesetters' treatments. The training must emphasize proper splinting procedures, criteria for hospital referral, adequate duration of healing, and simple rehabilitation tecniques. Second, there must be more dialogue established between the Jabari and government healthcare services through multilateral committees, consisting of government healthcare officials, Jabari, and community members. These committees could provide a forum for discussion on the importance of evidence-based practice, oversight in maximizing patient safety and outcomes, and building trust between all parties.

A key limitation of this study was that it may underestimate the dependence on Jabari for health care, as the study only obtained community member perspectives from the city of Am Timan. Most of the Salamat Region population lives in rural communities with minimal access to government healthcare services; therefore, dependence upon Jabari for all forms of healthcare is even more likely in rural communities. In addition, the scope of practice of the Jabari and their communities' relationships with them may vary across the different regions of Chad. The physicians were interviewed after the Jabari, which given our initial interview guide, this meant we were unable to ask follow-up questions with the Jabari, such as if they treat complications from fractures previously treated by physicians. Furthermore, the interview guide was not able to be back-translated by an independent translator, and the thematic content analysis was not completed in the participants' own languages. Other limitations include the underrepresentation of women in this study and the low sample sizes of Jabari and physicians. Limited demographic data was collected to protect identities given the controversial subjects discussed. Additionally, the physicians interviewed were not orthopedic specialists, therefore their views on trauma care may not be wholly representative of orthodox trauma care. More research is necessary to validate the findings of this exploratory study and further examine the Jabari's full scope of practice, community perspectives on trauma care, and interrelationships between the Jabari, government healthcare systems, and the community.

## Conclusion

Our research in Am Timan, Chad, analyzed the scope of Jabari practices and the relationships between Jabari, local physicians, and the community. Similarly to TBS in other African countries, community members often preferred Jabari due to their lower prices, their perceived efficacy in MSK injury treatment, and their conscientiousness and continuity of care for patients. However, we found that the Jabari's scope of practice far exceeds traumatic MSK injuries, which is substantially different from other bonesetters studied in sub-Saharan Africa. To reduce dangerous procedures and improve treatment, the authors recommend collaboration and training between Jabari and the physicians in Am Timan. Training the Jabari with orthopedists and opening channels of discourse between Jabari and medical officials are important steps in integrating Jabari into the Chadian healthcare system. Furthermore, the physicians could learn from Jabari in improving trust and patient-centered care with the local community. These interventions have the potential to greatly improve trauma care in Chad and promote trust and respect between the whole medical community and the people they serve.
